# Effects of health literacy competencies on patient-centered care among nurses

**DOI:** 10.1186/s12913-022-08550-w

**Published:** 2022-09-19

**Authors:** Yaki Yang

**Affiliations:** Department of Nursing, College of Medicine, Wonkwang University, Iksan, South Korea

**Keywords:** Health literacy, Clinical competency, Patient-centered care, Nurses

## Abstract

**Background:**

This study aims to identify the relationship between health literacy competencies and patient-centered care by clinical nurses.

**Methods:**

The participants were 180 nurses working at three university hospitals located in G City and J Province, South Korea. Self-evaluation questionnaires were used to collect data that were collected from June 1 to 30, 2021. Data were analyzed using descriptive statistics, independent t-test, one-way ANOVA, Pearson correlation coefficients, and multiple regression with SPSS 26.0.

**Results:**

The mean of health literacy competencies was 3.19 (4 point scale) and the mean of patient-centered care was 3.48 (5 point scale). There were significant positive relationships between health literacy competencies and patient-centered care by clinical nurses (*r* = .50, *p* < .001). Factors influencing the health literacy competencies of clinical nurses were identified as education level (university) (β = .82), education level (masters) (β = .74), prior health literacy knowledge (β = .52), and health literacy competencies (β = .44). The explanatory power of this regression model was 36%, which was statistically significant (*F* = 17.65, *p* < .001).

**Conclusion:**

Clinical nurses’ health literacy competencies should be developed to improve patient-centered care. Nursing education programs should emphasize the integration of health literacy into the nursing school curriculum.

## Background

Recently, as patients are being recognized as active consumers of medical services, their right to know has evolved, medical information has become popular, and medical service utilization has increased. In line with this, the paradigm of medical services has shifted from expert-to patient-centered [1, 2]. This also affects nursing care practice, and modern nursing care emphasizes on providing holistic patient-centered care that recognizes patients as individuals with personal needs [3]. Patient-centered care, also known as person-centered nursing or individualized nursing care, refers to providing routine nursing care, such as medication nursing according to a doctor’s prescriptions, assisting in health-related decision-making, and providing individualized care while considering the individual characteristics and needs of patients [4, 5].

Patient-centered care refers to respecting patients and focusing on their physical, psychological, social, and spiritual needs [6]. From a patient’s perspective, it has positive effects such as improved physical and mental functions and autonomy, which may result in shortening the length of hospital stay, reducing readmission and emergency room visits, and reducing mortality [7–9]. From the nurses’ perspective, patient-centered care can contribute to improving the continuity and quality of nursing care through nurses’ job satisfaction and organizational commitment [8, 10, 11]. Nurses’ competencies that affect patient-centered care include empathy, self-leadership, communication skills, nursing professionalism, prioritization, and management [12–15]. Since the American Medical Association defined patient-centered care, tools for its measurement, involving various elements, have been developed [16], and intervention studies have also been reported [17]. Since most health-related information contains technical content and difficult medical terms, patients may have difficulty communicating with medical professionals and acquiring health information. Therefore, to provide patient-centered care, communication between patients, their families, and healthcare providers with a focus on patients’ values, preferences, and needs is essential, and effective communication should be achieved in a way that patients can understand [13].

Health literacy refers to the ability to understand and acquire basic information and services for health-related decision making. Since it is related to all clinical situations, nurses should identify it in patients and be aware of its effects on patient outcomes [18]. Patients’ low health literacy can lead to negative consequences, such as lack of health-related knowledge, inadequate management of chronic diseases, poor medication adherence, increased emergency room visits, and increased hospitalization and mortality [19–21]. Failure to consider patients’ health literacy in communicating with them can have a profound impact on their ability to access and comply with healthcare services. If patients cannot understand medical instructions, it is difficult to expect them to take medications or practice healthy behaviors. However, because most healthcare workers lack the knowledge, skills, and attitude to effectively provide health information and health services to patients and their caregivers with low health literacy, that is, health literacy competencies, its training is emphasized [22]. It has been reported that only a few healthcare professionals use formal assessment tools to determine their patients’ health literacy, and the remaining majority use informal methods, such as their assumption or intuition, to roughly assess patients’ health literacy on the basis of patients’ education level [23]. Nurses are responsible for understanding various health information, such as management of patients’ hospital stay, medications and health education, and daily life and exercise after discharge, and they play an important role in promoting patients’ health literacy [24]. Therefore, nurses’ health literacy competencies in communicating with patients and providing patient education are professional nursing practice standards and the main competencies that nurses should have.

Nurses’ health literacy competencies can be the cornerstone of patient-centered care. This is because their failure to accurately assess patients’ health literacy may result in one-sided communication and limited effectiveness of patient education [25]. Therefore, many organizations recommend a ‘universal precaution’ approach [26, 27]. Enhancing health literacy is not a patient’s personal issue; thus, a comprehensive approach from the perspectives of health care providers and organizations should be made to form partnerships with patients and provide patient-centered care. Recently, medical curricula dealing with patients’ and physicians’ health literacy competencies in the medical science field are being actively provided, but such efforts in the nursing science field are insufficient. According to a study analyzing the educational goals of 60 nursing education institutions in South Korea, health literacy competencies were not included in nursing students’ expected abilities [28]. To develop curricula for improving nurses’ health literacy competencies, studies should determine health literacy competencies expected from nurses and identify priority factors to be conducted in advance as groundwork [18, 22].

Studies on health literacy in nursing science have, so far, mainly focused on specific patient groups and disease states, and nursing professionals’ educational preparations for health literacy competencies, the effects of health literacy competencies on nurses’ roles, and patient outcomes have not been evaluated [23, 29]. Some studies have reported significant differences between health literacy competency-related knowledge, skills, and practice among nurses [23, 30, 31]. Considering that nurses are responsible for direct patient care and delivery of medical services, it is necessary to determine their understanding and perception of health literacy competencies and to provide empirical evidence for their effects on patient-centered care. Therefore, this study aims to identify the association between health literacy competency levels and patient-centered care among nurses, present evidence for enhancing nursing care practice, and provide basic data for the composition of education programs on health literacy competencies.

It also aims to investigate the effects of health literacy competencies on patient-centered care among nurses and to understand the association between them.

## Methods

### Research design

This descriptive study aims to examine health literacy competencies and patient-centered care levels among nurses, and to determine their association.

### Participants

The participants in this study were nurses working at three university hospitals located in G City and J Province, South Korea, who understood the purpose and agreed to voluntarily participate. Participants were recruited by posting a notice on the bulletin board of the hospitals. New nurses with less than three months of clinical experience were excluded from this study, considering that they were focused on acquiring work-related knowledge and skills. The sample size was calculated with multiple regression analysis using the G*power 3.1 program [32]. The minimum number of samples required for this study were calculated to be 172 based on a significance level α of .05, power of 95%, medium effect size of .15, and 10 independent variables. Considering a dropout rate of approximately 10%, 200 questionnaires were distributed, of which 190 were collected. A total of 180 questionnaires were analyzed, excluding 10 with incomplete information.

### Measurement

#### Health literacy competencies

Health literacy competencies were measured using those [18] modified and supplemented from the ones in the clinical nurses scale [33]. This scale consists of three sub-domains with a total of 48 items, including 22 items regarding knowledge, 14 regarding skills, and 12 regarding attitude. This scale was measured on a 1–4 anchored scale, with higher scores indicating higher health literacy competencies.

The original author wrote the orthogonal rotation method with KMO and Kaiser regularization for construct validity analysis at the time of tool development. Exploratory factor analysis was performed using varimax. The KMO value was .93, each factor explained 12.8–16.8% of health literacy competencies, and the total cumulative explanation rate was 44.1% [33]. The reliability of the scale for all the items at the time of its development was Cronbach’s α = .95, and for the subdomains was Cronbach’s α = .91 for knowledge, Cronbach’s α = .89 for skills, and Cronbach’s α = .91 for attitude. The reliability of the tool for all items in this study was Cronbach’s α = .93, and for the subdomains was Cronbach’s α = .86 for knowledge, Cronbach’s α = .89 for skills, and Cronbach’s α = .90 for attitude.

#### Patient-centered care

Patient-centered care was measured using the Korean version of the Individualized Care Scale (ICS-nurses-A) [34], which was modified and translated from the original Individualized Care Scale (ICS-nurses-A) [11]. This tool consists of three subdomains with a total of 17 items, including seven regarding clinical situations, four regarding personal life situations, and six regarding decisional control. Each item is rated on a 5-point Likert scale, with a higher score indicating higher patient-centered care. A clinical situation is the process by which a nurse assesses and collects information about a patient’s preferences, needs, and perceptions. Personal life situations involve planning activities according to the patient’s characteristics, situational information, and response to health problems. Finally, decisional control allows patients to participate in the care according to their individual expectations and encouragement.

The original author wrote the orthogonal rotation method with KMO and Kaiser regularization for construct validity analysis at the time of tool development. Exploratory factor analysis was performed using varimax. The KMO value was .91, three factors with an Eigen value of 1.0 or higher were extracted, and the factor load value of the item was in the range of .45–.80. Each factor explained 6.5–36.3% of patient-centered nursing, and the total cumulative explanation rate was 52.1% [11].

The reliability of the ICS-nurses-A at its development was Cronbach’s α = .80 for all the items, with Cronbach’s α = .83 for clinical situation, Cronbach’s α = .70 for personal life situation, and Cronbach’s α = .72 for decisional control. The reliability of the Korean version of the ICS-nurses-A in this study was Cronbach’s α = .94 for all the items, with Cronbach’s α = .87 for clinical situation, Cronbach’s α = .88 for personal life situation, and Cronbach’s α = .89 for decisional control.

### Data collection and ethical approval

The data collection period was from June 1, 2021 to June 30, 2021. Before data collection, this study was approved by the institutional ethics committee (No. WK201901-SB-005) of the institution to which the researcher belongs. This study was conducted in accordance with the Declaration of Helsinki. Informed consent was obtained from all the subjects. Data were collected using self-reporting questionnaires. The purpose and procedure of this study were explained to the participants prior to data collection. To protect their rights, data were collected after they signed the written consent form. For the participants to answer the questionnaire honestly, the consent form for this study was printed and distributed among the participants so that each participant could sign it and submit it in a sealed return envelope. Participants were informed in advance that all completed data would be anonymously coded, destroyed at the end of this study, and never be used for any purpose other than the purpose of this study.

### Data analysis

The collected data were analyzed using SPSS 26.0. The detailed data analysis methods are as follows.

- The participants’ general characteristics, health literacy competencies, and patient-centered care levels were analyzed based on descriptive statistics.
- The differences in mean scores in patient-centered care levels according to the general characteristics of the subjects were analyzed using independent t-test and one-way ANOVA.
- The correlations between health literacy competencies and patient-centered care levels using mean ratings were analyzed using Pearson’s correlation coefficient.
- Factors affecting patient-centered care levels were analyzed using multiple regression analysis.

## Results

### Demographics

A total of 180 participants were included, and 96.7% of them were female. In terms of age, the proportion of those aged 30 years or younger was highest at 45%. In terms of marital status, 65% were unmarried. 71.7% had no religion. In terms of educational level, 81.7% were university graduates. In terms of job positions, 90.6% were general nurses. The proportion of those with 10–19 years of clinical experience was the highest at 31.1%. In terms of working departments, 30.6% worked in medical wards. In addition, 46.7% had experience in dealing with health literacy in nursing education courses or clinical situations (Table 1).

**Table 1:** Demographics of participants (*n* = 180)

Characteristics	Categories	N(%)
Gender	Male	6 (3.3)
	Female	174 (96.7)
Age (year)	<30	81 (45.0)
	30–39	69 (38.3)
	≥40	30 (16.7)
Marital status	Unmarried	117 (65.0)
	Married	63 (35.0)
Education level	Diploma^a^	3 (1.7)
	Bachelor^b^	147 (81.7)
	≥Master^c^	30 (16.6)
Religion	Yes	51 (28.3)
	No	129 (71.7)
Job position	General nurse	163 (90.6)
	Staff nurse	17 (9.4)
Total clinical experience (year)	<2	29 (16.1)
	2–4	44 (24.4)
	5–9	39 (21.7)
	10–19	56 (31.1)
	≥20	12 (6.7)
Working unit	Medical ward	55 (30.6)
	Surgical ward	11 (6.1)
	Intensive care unit	42 (23.3)
	Emergency room	43 (23.9)
	Operation room	29 (16.1)
Prior health literacy knowledge	Yes	84 (46.7)
	No	96 (53.3)

### Degrees of mean scores health literacy competencies and patient-centered care

The mean score for health literacy competencies among the participants was 3.19 ± 0.29 points (out of 4 points). By its sub-domain, the mean score for attitudes was the highest with 3.39 ± 0.42 points, followed by 3.15 ± 0.29 points for knowledge and 3.08 ± 0.40 points for skills. The mean score for patient-centered care was 3.48 ± 0.69 (out of 5 points). By its subdomain, the mean score for clinical situation was the highest with 3.61 ± 0.62 points, followed by 3.52 ± 0.76 points for decisional control and 3.20 ± 0.92 points for personal life situation (Table 2).

**Table 2:** Degrees of mean scores health literacy competencies and patient-centered care (*n* = 180)

Variables	Sub-domain	Mean ± SD	Min ∼ Max	Range
Health literacy competencies	Knowledge	3.15 ± 0.29	2.55 ∼ 3.86	1 ∼ 4
	Skill	3.08 ± 0.40	2.21 ∼ 4.00	1 ∼ 4
	Attitude	3.39 ± 0.42	2.42 ∼ 4.00	1 ∼ 4
	**Total**	**3.19 ± 0.29**	**2.44 ∼ 3.94**	**1 ∼ 4**
Patient-centered care	Clinical situation	3.61 ± 0.62	2.44 ∼ 3.94	1 ∼ 5
	Personal life situation	3.20 ± 0.92	1.00 ∼ 5.00	1 ∼ 5
	Decisional control	3.52 ± 0.76	1.17 ∼ 5.00	1 ∼ 5
	**Total**	**3.48 ± 0.69**	**1.47 ∼ 5.00**	**1 ∼ 5**

### Patient-centered care according to the general characteristics of the participants

The results of investigating the differences in patient-centered care according to the general characteristics of the participants revealed significant differences in the care according to religion, education level, total clinical experience, and prior health literacy knowledge. The results of a post-hoc analysis revealed that university graduates and those with a master’s degree or higher had higher patient-centered care scores than college graduates (Table 3).

**Table 3:** Patient-centered care according to the general characteristics of the participants (*n* = 180)

Characteristics	Categories	Mean ± SD	t/F	*p*	Scheffé
Gender	Male	3.69 ± 0.53	0.74	.460	
	Female	3.47 ± 0.68			
Age (year)	<30	3.59 ± 0.71	2.10	.126	
	30–39	3.37 ± 0.65			
	≥40	3.46 ± 0.64			
Marital status	Unmarried	3.47 ± 0.72	−0.49	.624	
	Married	3.52 ± 0.60			
Education level	Diploma^a^	1.94 ± 0.82	9.25	<.001	a < b,c
	Bachelor^b^	3.54 ± 0.68			
	≥Master^c^	3.39 ± 0.47			
Religion	Yes	3.65 ± 0.70	2.06	.041	
	No	3.42 ± 0.66			
Job position	General nurse	3.50 ± 0.69	−1.13	.261	
	Staff nurse	3.31 ± 0.51			
Total clinical experience (year)	<2	3.74 ± 0.78	3.21	.014	–
	2–4	3.59 ± 0.68			
	5–9	3.34 ± 0.57			
	10–19	3.32 ± 0.62			
	≥20	3.75 ± 0.74			
Working unit	Medical ward	3.55 ± 0.70	1.47	.212	
	Surgical ward	3.50 ± 0.54			
	Intensive care unit	3.61 ± 0.62			
	Emergency room	3.43 ± 0.75			
	Operation room	3.25 ± 0.63			
Prior health literacy knowledge	Yes	3.67 ± 0.57	3.53	.001	
	No	3.32 ± 0.73			

### Correlation between health literacy competencies and patient-centered care

The results of analyzing the correlation between health literacy competencies and each subdomain (health literacy knowledge, skills, and attitude) and patient-centered care revealed that the competencies had a positive correlation with the care (*r* = .50, *p* < .001). Each of the subdomains of health literacy knowledge, skills, and attitude had a positive correlation with patient-centered care (*p* < .001) (Table 4).

**Table 4:** Correlations of Variables (*n* = 180)

Variables	Knowledge	Skill	Attitude	Health literacy competencies
	r (*p*)	r (*p*)	r (*p*)	r (*p*)
Patient-centered care	.35 (<.001)	.58 (<.001)	.30 (<.001)	.50 (<.001)

### Factors affecting patient-centered care

Multiple regression analysis was performed to identify the factors affecting patient-centered care. Religion, education level, total clinical career, and prior health literacy knowledge, which showed a significant difference in patient-centered care among general characteristics, were used as independent variables. Health literacy competencies were also added. Religion, educational level, and prior health literacy knowledge were treated as dummy variables.

Collinearity is the relationship between two independent variables. If the correlation coefficient between two independent variables is 1, it means perfect collinearity; if it is 0, it means there is no collinearity at all. The relationship between three or more variables is known as multicollinearity. To check this more strictly, we checked variance inflation factor (VIF). The higher the VIF value, the higher is the collinearity. In general, if it is 10 or less, there is no collinearity problem. The results of calculating multicollinearity between the independent variables confirmed that there was no multicollinearity because the VIF was less than 10. The analysis revealed a statistically significant association between health literacy competencies (β = .44, *p* < .001) and patient-centered care. Higher health literacy competencies were associated with higher patient-centered care. The regression model used in this study was statistically significant (*F* = 17.65, *P* < 0.01). The overall explanatory power of the model was 36% (Table 5).

**Table 5:** Factors affecting patient-centered care (*n* = 180)

Variable	B	SE	β	t	*p*
(Constant)	−1.17	.54		−2.17	.031
Religion^a^ (Yes)	0.16	.09	.11	1.72	.087
Education level^a^ (university)	1.44	.32	.82	4.43	<.001
Education level^a^ (master)	1.34	.33	.74	4.03	<.001
Total clinical experience	−0.06	.04	−.10	−1.52	.130
Prior health literacy knowledge^a^ (Yes)	0.54	.12	.52	4.66	<.001
Health literacy competencies	1.02	.15	.44	6.96	<.001

R^2^ = .38, Adjusted R^2^ = .36, F = 17.65, *p* < .001

*SE* Standard error, *Adj* Adjusted

^a^Dummy variables

## Discussion

This study attempted to investigate health literacy competencies and patient-centered care levels, and to determine the association between health literacy competencies and patient-centered care among nurses. The following interpretations were made based on the results of this study.

Of the 180 participants in this study, 46.7% had experience dealing with health literacy in nursing education courses or clinical situations, which was lower than the 80.0% found in a study involving nurses in the US [23], and higher than the 38.9% found in a study involving nurses in Iran [31]. Most studies on health literacy in South Korea have focused on patients’ health literacy, but not on medical professionals’ health literacy competencies. There is also a lack of programs on health literacy competencies in nursing education. These findings suggest that nurses may have difficulty understanding the reality of patients’ health literacy competencies and related problems.

The scores for health literacy knowledge, skills, and attitude, which are the subdomains of health literacy competencies, were 3.15, 3.08, and 3.39 (out of 4 points), respectively, indicating that the scores for knowledge and attitude were higher compared to the score for skills. With regard to knowledge, the score for the item: ‘I know the red flag behaviors suggesting that a patient lacks the ability to understand health information’ was the lowest with 2.72 points, and the mean score for the item: ‘I am aware of the difference between reading ability and reading comprehension ability, and the reason why general reading skills do not guarantee a patient’s understanding’ was low with 2.83 points.

With regard to attitude, the mean score for the item ‘universal precautions’ approach for all patients is required because it is difficult to distinguish persons at the risk of communication errors through simple observations, and the ordinary patient-caregiver interactions’ was low with (3.18 points. As found in previous studies [30, 35] reporting that most medical professionals tend to overestimate patients’ health literacy, nurses who participated in this study may also have overestimated patients’ health literacy when communicating with them. In fact, since individual patients’ knowledge about medical terms and information needs may be inaccurately identified and their health literacy overestimated, there is a possibility that communication might be conducted in a way that patients cannot understand well. According to a study analyzing the educational goals of 60 nursing education institutions in South Korea, health literacy competencies were not included in nursing students’ expected abilities [28]. There is a need to establish educational goals related to nurses’ health literacy competencies in universities and medical institutions to compose a curriculum or provide additional educational programs in clinical practice. With regard to skills, the score for the item: ‘I can find out individual patients’ prior understanding level of health problems without making them feel ashamed’ was the lowest at 2.81 points. Hospitals generally provide patients with test guidance and handouts such as patient education materials in a textural format [36]. Efforts are required to identify patients’ cultural and social characteristics and understanding levels, and to provide appropriate education. The scores for the items: “I can accurately and effectively communicate verbally in patients’ preferred language using medical interpretation services’ and ‘I can provide resources for patients with disabilities’ were low at 3.01 points and 2.83 points, respectively. It has been reported that the biggest problems with medical services felt by foreigners in South Korea were a lack of medical services that took into account their cultural characteristics and communication difficulties [37], and that individuals with hearing and speech impairments had reduced medical service utilization and decreased satisfaction due to communication difficulties [38]. It is necessary to improve interpretation services so that patients and healthcare workers can use them 24 hours a day, and to provide proactive support to address the low health literacy of vulnerable social groups including disabled people.

In terms of the subdomain of patient-centered care, the score for clinical situation was the highest at 3.61 (out of 5 points), the score for decisional control was 3.52 (out of 5 points), and the score for personal life situation was the lowest at 3.20 (out of 5 points). These results are similar to the results of a study measuring patient-centered care in nurses using the same tool [14] and are consistent with the results of a study conducted in seven countries, including the US and Finland, in which the score for the clinical situation subdomain was the highest [39]. Most nurses prioritize identifying patients’ disease state when providing nursing care and perceive that it is important to identify patients’ needs in clinical situations and reflecting them in nursing care [40]. As the recent trend of medical services has changed from disease- to patient-centered, patient-centered communication has been emphasized as a factor that positively affects patient health outcomes [41]. However, for its active application, as in the US and European countries, sufficient communication training should be provided to nursing students, considering patients’ health literacy levels and clinical situations in nursing education curriculums at nursing colleges. The score for the subdomain personal life situation, which is supposed to reflect in nursing care by asking questions about individual patients’ hospitalization experience and daily life habits, was the lowest. For patient-centered care, it is important to provide patients with individualized nursing care in consideration of their personal life situations. However, in actual clinical settings, nurses only collect personalized data through a nursing information survey immediately after a patient’s hospitalization, and it is difficult to additionally assess the patient’s preferences and needs and to reflect them in nursing care due to the heavy workload. Although patient-centered care, which perceives patients as individuals with diverse and individual needs, is being established as a major paradigm [3], it can be said that under the current medical system, it is difficult to provide because of the lack of the required nursing workforce [42]. In order to consider patients’ personal aspects, it is necessary to improve nurses’ perceptions and attitudes, policies, and institutional efforts at the level of hospitals, and the government needs to relocate the nursing workforce and adjust the number of patients per nurse to reduce nurses’ workload and burden.

The results of this study showed significant differences in patient-centered care according to religion, education level, total clinical experience, and prior health literacy knowledge among the general characteristics of the participants, which differ from the results of previous studies indicating that there was a significant difference according to age and working department [14, 43]. Therefore, repetitive studies involving different participants and clinical situations are required to investigate the general characteristics of patient-centered care.

Health literacy competencies and their subdomains: knowledge, skills, and attitudes, all had a positive association with patient-centered care. The skills subdomain had the highest association with patient-centered care (*r* = .58), followed by knowledge (*r* = .35) and attitude (*r* = .30). Medical professionals should have different types of health literacy skills and the related practical principles. Therefore, studies are needed to prioritize the subdomains of health literacy competencies by identifying their subdomain [26] that have the greatest potential impact on patient outcomes and evaluating their relative values. Based on participants’ self-assessments in this study, the score for health literacy skills was relatively lower compared to the score for knowledge or attitude. Empirical teaching methods can be suggested to practice specific strategic approaches for communicating with patients with low health literacy competencies, such as changing technical terms into easier terms, drawing pictures, and checking patients’ understanding level using the teach-back technique [44, 45].

When multiple regression analysis was performed to identify the major factors affecting patient-centered care, the results revealed that higher health literacy competencies were positively associated with higher patient-centered care, its explanatory power was 36%. This finding supports the results of a study indicating that nurses’ health literacy knowledge, skills, and attitudes will be the basis of patient-centered care [46]. Inconsistent with the principles of universal precautions for health literacy, it was reported that it is an important process for realizing patient-centered care for nurses to identify individual patients’ health literacy and use differentiated communication strategies for patients with low health literacy [47]. Although the importance of patient-centered care is being emphasized in South Korea, interest and efforts in nurses’ health literacy competencies are lacking. To achieve patient-centered care, additional research is required to develop standards and practice guidelines for health literacy competencies for nurses to use tools to accurately identify patients’ health literacy or to communicate effectively according to its level reflecting the cultural context of South Korea; for example, Chinese characters are often used in medical terms. This study is significant in that it identified nurses’ health literacy competencies and determined their association with patient-centered care. In addition, the results of this study will be a basis for developing health literacy curricula to enhance health literacy competencies in nursing students and clinical nurses in the current local situation where health literacy practice is not consistently applied and guidelines for its content and structure are not established. It will also be a basis for improving the quality of nursing in providing patient-centered care.

## Conclusions

This study found that health literacy competencies among nurses had a high association with patient-centered care and confirmed that these competencies among nurses were a significant influencing factor for the care. Based on the results of this study, we suggest the following. First, because this study involved nurses from only three university hospitals located in G City and J Province in South Korea, it is difficult to generalize the results. Therefore, repetitive studies with different sample sizes and clinical situations are required in the future. Second, since studies on the importance and priority of health literacy competencies among nurses are scarce, it is necessary to determine their importance and priority as perceived by nurses and patients and to develop curricula their improvement and programs for their strengthening among nursing students. Third, policy support such as improving access to medical interpretation services and developing various educational materials should be provided to improve patients’ health literacy. Policy improvements, such as nursing workforce relocation and adjusting the number of patients per nurse, are suggested to reduce nurses’ workload and burden in providing patient-centered care.

## Limitations

The measurement tools relied on self-reported patient-centeredness and health literacy knowledge, skills, and attitudes. Self-reported data are susceptible to a social desirability bias. In addition, studies examining the self-assessed communication skills of healthcare professionals suggest that they are often poor at self-assessment of such skills. An observational study would potentially provide greater validity for the results. This study did not use a theoretical model to propose a relationship between patient-centered care and health literacy competencies. Having a theoretical model could improve the ability to empirically test the potential relationships between them.

The findings of this study were correlational. The design did not provide evidence for the direction of causality. We do not know whether stronger self-reported health literacy competencies cause stronger self-reported patient-centered care, or vice versa. As such, we do not know whether teaching nurses about one of these constructs will improve their self-ratings of the other. Further prospective studies are required to determine the direction of causality.

## Data Availability

The datasets during and / or analysed during the current study available from the corresponding author on reasonable request.
